# Association of small vessel disease with tau pathology

**DOI:** 10.1007/s00401-021-02397-x

**Published:** 2022-01-19

**Authors:** Alifiya Kapasi, L. Yu, V. Petyuk, K. Arfanakis, D. A. Bennett, J. A. Schneider

**Affiliations:** 1 Rush Alzheimer’s Disease Center, Rush University Medical Center, 1750 W Harrison Street, Chicago, IL 60612 USA; 2 Department of Pathology, Rush University Medical Center, Chicago, IL USA; 3 Department of Neurological Sciences, Rush University Medical Center, Chicago, IL USA; 4 Biological Sciences Division, Pacific Northwest National Laboratory, Richland, WA USA; 5 Department of Diagnostic Radiology and Nuclear Medicine, Chicago, IL USA; 6 Department of Biomedical Engineering, Illinois Institute of Technology, Chicago, IL USA

**Keywords:** Small vessel disease, Tau pathology, Neuropathology, Aging

## Abstract

Emerging evidence suggests that small vessel disease (SVD) is a risk factor for clinical dementia and may contribute to AD neuropathological changes. Watershed brain regions are located at the most distal areas between arterial territories, making them vulnerable to SVD-related changes. We examined the association of pathologic markers of SVD, specifically arteriolosclerosis in watershed brain regions, with AD pathologic changes. Participants (*N* = 982; mean age-at-death = 90; 69% women) were enrolled as part of one of two cohort studies of aging and dementia. At autopsy, neuropathological evaluation included semi-quantitative grading of arteriolosclerosis pathology from 2 cortical watershed regions: the anterior watershed (AWS) and posterior watershed (PWS), densities for cortical β-amyloid and tau-tangle pathology, and other common age-related pathologies. Linear regression models examined the association of watershed arteriolosclerosis pathology with β-amyloid and tau-tangle burden. In follow-up analyses, available ex-vivo MRI and proteomics data in a subset of decedents were leveraged to examine the association of whole brain measure of WMH, as a presumed MRI marker of SVD, with β-amyloid and tau-tangle burden, as well as to examine the association of watershed arteriolosclerosis with proteomic tau. Watershed arteriolosclerosis was common, with 45% of older persons having moderate-to-severe arteriolosclerosis pathology in the AWS region, and 35% in the PWS. In fully adjusted models that controlled for demographics and common age-related pathologies, an increase in severity of PWS arteriolosclerosis was associated with a higher burden of tau-tangle burden, specifically neocortical tau burden, but not with β-amyloid. AWS arteriolosclerosis was not associated with β-amyloid or tau pathology. Ex-vivo WMH was associated with greater tau-tangle pathology burden but not β-amyloid. Furthermore, PWS arteriolosclerosis was associated with higher abundance of tau phosphopeptides, that promote formation of tau aggregates. These data provide compelling evidence that SVD, specifically posterior watershed arteriolosclerosis pathology, is linked with tau pathological changes in the aging brain.

## Introduction

Cerebral small vessel disease (SVD) is one of the most common causes of vascular cognitive impairment and dementia. SVD is an umbrella term which refers to the spectrum of pathological changes affecting small vessels (arteries, arterioles, venules, and capillaries), as well as the diverse vascular tissue injuries impacting both gray and white matter structures in the brain [11, 17, 18]. There has been longstanding recognition of vascular dysfunction in multiple neurodegenerative diseases, particularly Alzheimer’s disease [8, 26, 40, 46, 60, 68]. We and others have shown that majority of older persons diagnosed with Alzheimer’s dementia have one or more vascular pathologies, highlighting a central role of cerebrovascular disease in cognitive aging pathogenesis. Complicating matters further, a large proportion of older persons (80%) have co-existing neurodegenerative proteinopathies with vascular pathology, with the most common being AD mixed with vascular pathologic changes [25, 27]. Despite this large burden of comorbidity in the aging brain, the pathobiologic interplay between vascular and neurodegenerative mechanisms remains poorly understood.

Cerebral Aβ plaques and pathologic tau indicate specific neuropathologic changes that define AD pathology. There is a longstanding literature examining the association between SVD with β-amyloid [22, 38, 63, 67], with more recent studies suggesting a refined relationship between SVD and tau [10, 32, 39, 51, 70]. However, much of the evidence for the latter is derived from animal models, with limited data from human-based studies. One hypothesis suggests that small vessel disease may accelerate the pathological process underlying AD via chronic cerebral hypoperfusion. Notably, cerebral hypoperfusion is a well-established phenotype in patients with AD, a predictor of cognitive decline, and emerging as a candidate biomarker for dementia risk [15, 21]. While it remains unclear whether hypoperfusion in AD is a cause or consequence of disease pathogenesis, accumulating evidence suggests an intertwined relationship between cerebral blood flow, β-amyloid, and tau.

Watershed border zones are located between two or more arterial territories and supplied by the most terminal arterioles in the brain. We and others have shown that these brain regions are particularly vulnerable to hypoperfusion, vasculature changes, and ischemic-tissue injury [29, 37, 61]; thus, making watershed brain regions ideal to shed light on the role of the vasculature in AD. In this study, we leverage neuropathologic, ex-vivo MRI, and proteomic data to systematically examine the association between SVD and AD pathological changes.

## Methods

### Participants

**Fig. 1:**
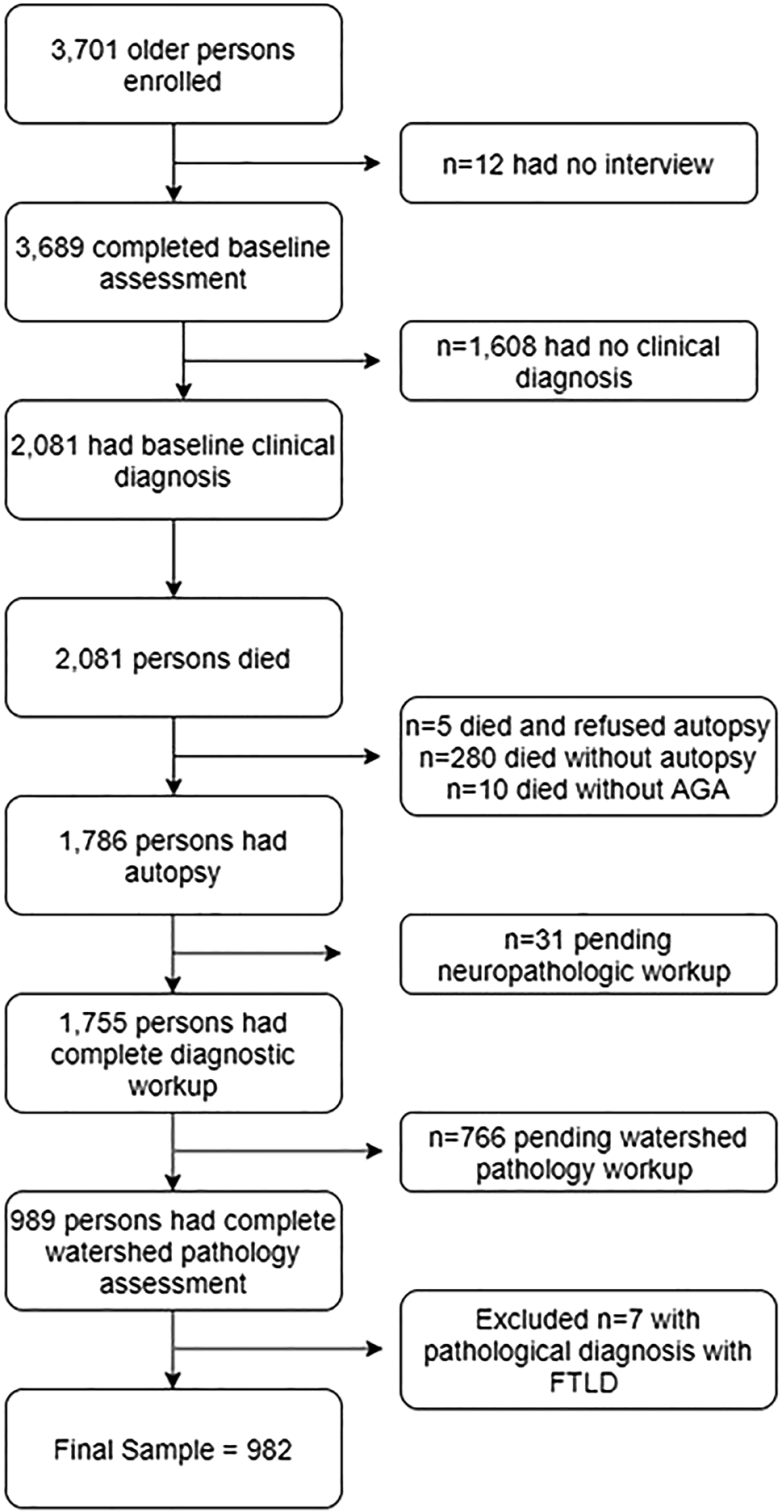
Study sample

Participants enrolled in the Religious Orders Study (ROS) and the Rush Memory and Aging Project (MAP). Eligibility in both studies requires enrollment without known dementia, and agreement to undergo annual clinical evaluation and interview. Each study was approved by the Rush University Medical Center institutional review board [9]. Participants in both ROS and MAP signed an informed consent, and an Anatomical Gift Act for brain donation at the time of death. Follow-up across all studies exceeding 85%, and autopsy rates exceeding 95% for ROS and 80% for MAP. At the time of the analyses, 2081 older persons died and 1786 were autopsied. Complete neuropathologic workup for diagnostic purposes was evaluated on 1755, and of those, 989 persons had complete watershed arteriolosclerosis pathology assessment. We excluded 7 FTLD/tauopathy cases, leaving a final sample size of 982 (Fig. 1). Date of birth (to calculate age), sex, and education (in years) are reported at baseline.

### Vascular risk factors/diseases

Diabetes was present if the participant was ever-taking medication to treat diabetes of if there was history of diagnosis of diabetes. Hypertension was based on whether participant had a history of hypertension. Smoking was reported as participant was a former or current smoker. Overall vascular risk burden score was quantified as ‘ever present’ if participant ever reported diabetes, hypertension, or smoking during the study. Additional variables related to blood pressure were also measured at baseline and annually. Participants competed three blood pressure reading while seated and standing. Systolic and diastolic blood pressures were calculated by averaging all three readings. Vascular disease burden was present if participants self-reported the presence of claudication, stroke, or myocardial infarction during the study. Analyses included the overall vascular risk burden, the mean of all average systolic/diastolic blood pressures taken across all visits, and the overall vascular disease burden.

### Neuropathology

Brain autopsies were performed following a standard protocol, as previously described [57]. Average post-mortem interval was 9.5 h (SD = 7.81). One hemisphere was cut into 1-cm coronal slabs and frozen. The dorsolateral prefrontal cortex from the frozen hemisphere was used for generating proteomic data. The contralateral hemisphere was cut into 1-cm coronal slabs and fixed in 4% paraformaldehyde. Paraformaldehyde fixed tissue from multiple brain regions was collected for various pathology data collection purposes, as described below.

#### Watershed arteriolosclerosis

**Fig. 2:**
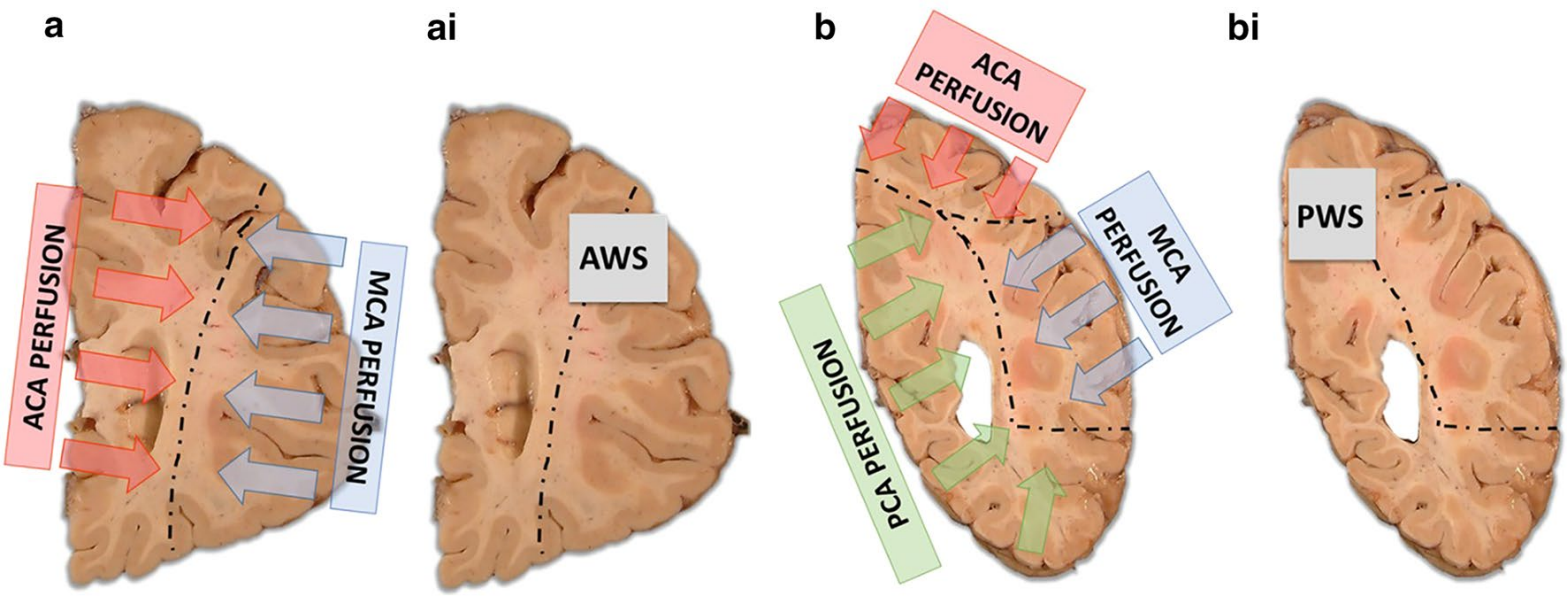
Schematic representation of watershed border zones and tissue sampling. A schematic representation of the anterior, middle, and posterior cerebral arterial territories marked by the dotted lines, highlighting areas of the anterior and posterior watershed border zones

**Fig. 3:**
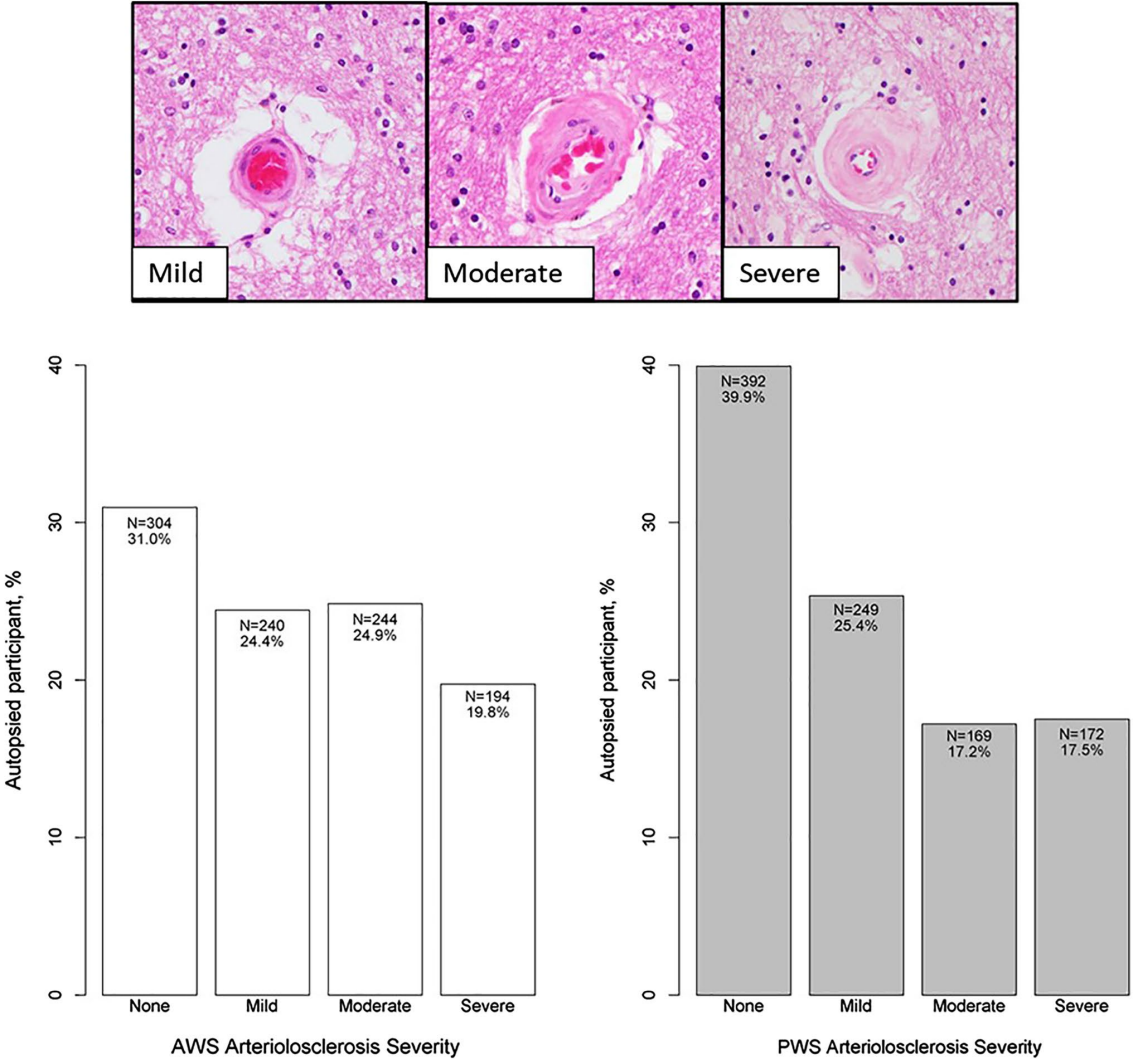
Watershed arteriolosclerosis pathology. Bar chart demonstrating the severity of arteriolosclerosis pathology in AWS and PWS regions

Tissue from two regions were collected as representative watershed areas; the subcortical frontal white matter overlying the ACA–MCA border zone, which we termed as ‘anterior watershed (AWS)’, and the parieto-occipital region lying at the ACA–MCA–PAC border zone, termed as ‘posterior watershed (PWS)’ (Fig. 2). Standardized protocols were followed for tissue sampling. The anterior watershed block was consistently taken deep to the midfrontal gyrus from the 1st slab anterior to caudate and putamen. The posterior watershed block is a region that lies within the precuneus and taken 1 or 2 slabs posterior to the end of the hippocampus and taken inferior-medial to Brodmann Area (BA) 7. Small vessels within the white matter in both AWS and PWS regions were evaluated on hematoxylin and eosin stained sections for arteriolosclerosis severity. Grading used a semi-quantitative 4-level rating system (0 = none, 1 = mild, 2 = moderate, and 3 = severe) based on the histological changes of the small arterioles, including intimal deterioration, degeneration of smooth muscle cells, and hyaline concentric thickening associated with vascular lumen narrowing. (Fig. 3). Two separate raters evaluated the severity of arteriolosclerosis pathology. The weighted kappa statistics for the inter-rater agreement was 0.79, reflecting excellent agreement.

#### Cortical β-amyloid load and PHF-tau-tangle density

Tissue blocks from eight cortical brain regions; entorhinal, midfrontal [Brodmann (BA) 46/9], inferior temporal (BA 20), inferior parietal (BA 39/40), calcarine (BA 17), anterior cingulate (BA 24), and superior frontal (BA 6/8) cortices, and CA1/subiculum subfield of the hippocampus, were cut into 20 µm sections. For assessment of cortical β-amyloid, immunohistochemistry was performed using three monoclonal antibodies against Aβ, 4G8 (1:9000; Covance Labs, Madison, WI), 6F/3D (1:50; Dako North America Inc., Carpinteria, CA), and 10D5 (1:600; Elan Pharmaceuticals, San Francisco, CA). For amyloid load quantification, raters outlined the cortex excluding the leptomeninges, and applied a custom positive-pixel algorithm developed by ImageJ software. This amyloid load consisted of cortical amyloid plaques and parenchymal vessels which contain amyloid. Value was calculated as percent area of cortex occupied by amyloid-beta deposits, as previously described [28]. For assessment of tau-tangle pathology, an antibody specific to phosphorylated tau, AT8 (1:2000, Thermoscientific) was used. Quantification of tangle density per square millimeter was performed by stereological mapping software by experienced raters. For analyses, a composite measure for overall β-amyloid burden and overall PHF-tau-tangle density was obtained by averaging the mean percentage area per region, across all regions [28]. In addition, a mesial temporal composite (across hippocampus and entorhinal regions) and neocortical composite burden for both β-amyloid and tau tangles was obtained.

#### Other cerebrovascular pathologies

Three additional measures of cerebrovascular disease pathologies included: *Atherosclerosis*—Large vessel at the circle of Willis at the base of the brain were evaluated visually using a semi-quantitative 4-level grading system (none, mild, moderate, and severe). None was graded when no significant atherosclerosis was present, mild was graded when less than 25% of vessel was involved, moderate was graded when atherosclerosis was present in 50% of all major vessels or if a single vessel had 50% occlusion, and serve was graded when atherosclerosis was present in more than 50% of vessels of if one or more vessels had 75% occlusion [5]. *Cerebral Amyloid Angiopathy (CAA)*—Meningeal and parenchymal vessels from 4 neocortical regions were evaluated by experience neuropathologists on sections immunostained with monoclonal antibodies against Aβ. CAA was evaluated by experienced neuropathologists and were scored as 0 (no amyloid deposition was seen in vessels), 1 (vessels had scant amyloid deposition), 2 (up to 10 vessels had circumferential amyloid deposition), 3 (50–75% of vessel have circumferential amyloid deposition, and 4 (when > 75% of vessels have amyloid). A summary score for each region was created using the maximum of the meningeal and parenchymal score. A continuous summary score was created by averaging scores across regions [13]. *Infarcts—*Macroscopic infarcts were identified visually on gross examination and age of infarct was confirmed by histology. Microscopic infarcts were not visible to the naked eye and identified by microscopy in tissue blocks. Location and age were also documented [6, 55]. For analyses, only chronic infarcts were considered, and all infarct variables were categorized into absent vs. present.

#### Other neurodegenerative pathologies

Two additional measures of neurodegenerative pathologies included: *LATE Neuropathologic Change (LATE-NC)*—Phosphorylated transactive response DNA-binding protein 43 (TDP-43) pathology was assessed by immunohistochemistry using a monoclonal antibody to phosphorylated TDP-43 (pS409/410; 1:100) in eight brain regions (amygdala, CA1, dentate gyrus, entorhinal cortex, anterior temporal pole, midtemporal gyrus, middle frontal gyrus, and inferior orbital frontal). Semi-quantitative inclusion measures were determined into four distinct stages based on the distribution of TDP-43 lesions; stage 0 (no presence of TDP-43), stage 1 (TDP-43 localized to the amygdala), stage 2 (extension of TDP-43 to the hippocampus and/or entorhinal cortex), and stage 3 (extension into the neocortex) [45]. *Lewy Body Pathology*—Lewy bodies were identified with antibodies to phosphorylated α-synuclein. We used a modified version of the McKeith Criteria [41] to identify nigra_predominant as cases with Lewy bodies in the substantia nigra alone, limbic type as cases with Lewy body pathology extending into amygdala, entorhinal, or anterior cingulate cortices, and neocortical type as cases with Lewy body pathology extending into either midfrontal, temporal, or inferior parietal cortices [56].

### Ex-vivo WMH burden

At the time of autopsy, the hemisphere identified with the most pathology or least mechanical damage was immersed in phosphate-buffered 4% formaldehyde solution at 4 °C. The intact hemisphere while immersed in fixative solution was sent for MRI scanning. The hemisphere was positioned with the medial aspect facing the bottom of container. Ex-vivo MRI data were collected on 3 Tesla scanners using 2D fluid-attenuated inversion recovery (FLAIR) and 2D multi-echo spin-echo (ME-SE) sequences, as previously described [4]. All data were collected sagittally and were converted to the axial plane (trilinear interpolation was used). Intensity standardization approaches were applied for normalization across participants. In all participants, WMH burden based on ex-vivo FLAIR and ME-SE images used a modification of the original Fazekas approach. WMH burden was rated separately in periventricular and deep white matter according to the original four-level Fazekas scale. However, ratings of 0 and 1 were combined resulting in WMH burden scale with a 3-level scale: 1 (mild), 2 (moderate), and 3 (severe). The maximum of the periventricular and deep white matter ratings was used as the whole brain WMH rating. Intra-rater reliability for WMH burden rating was excellent (ICC = 0.75, *p* < 10–50) and agreement of the rater with the expert was good (ICC = 0.64, *p* = 3 × 10–5).

### Targeted proteomics: selected reaction monitoring (SRM)

Autopsy measures for tau phosphopeptides abundance were quantified using Quantitative SRM proteomics from frozen dorsolateral prefrontal tissue samples (gray matter) (BA 9).

#### Sample preparation

Samples were prepared for LC-SRM analysis using standard protocol described elsewhere [3, 50, 71]. An average ∼ 20 mg of brain tissue from each individual was homogenized in denaturation buffer (8 M urea, 50 mM Tris–HCl pH 7.5, 10 mM DTT, 1 mM EDTA). Next, protein aliquots (400 µg) were alkylated with 40 mM iodoacetamide and digested with trypsin Digests were cleaned using solid-phase extraction (SPE) using Strata C18-E (55 μm, 70 Å) 25 mg/well 96-well plates on positive pressure manifold CEREX96 (SPEware Corporation, Baldwin Park, CA). Peptide concentrations obtained were measured using a bicinchoninic acid assay. Tryptic peptide concentrations were readjusted to 1ug/ul.

#### SRM data

LC-SRM experiments were performed on a nano-ACQUITY UPLC coupled to a TSQ Vantage MS instrument, with 2 µl of sample injection for each measurement. Formic acid (0.1%) in water and 0.1% in 90% acetonitrile were used as buffer A and B, respectively. Peptide separations were performed by an ACQUITY UPLC BEH 1.7-µm C18 column (75 µm i.d. × 25 cm) at a flow rate 350 nl/min using gradient of 0.5% of buffer B in 0–14.5 min, 0.5% to 15% B in 14.5–15.0 min, 15% to 40% B in 15–30 min, and 45% to 90% B in 30–32 min.

Endogenous (light) peptide abundances were measured relative to the spiked-in stable isotope-labeled synthetic peptide standards (heavy). SRM data were analyzed by Skyline software [64-bit version (MacCoss Lab Software)], and were manually inspected to ensure correct peak assignment, transitions, and boundaries. The peak area ratios of endogenous light peptides and their heavy isotope-labeled internal standards (i.e., L/H peak area ratios) were then automatically calculated by the Skyline software, and the best transition without matrix interference was used for accurate quantification. Peptide ratios (light/heavy) were log2-transformed and centered at the median.

#### Tau phosphopeptides’ data

We selected 5 different known tau phosphorylation sites for targeted measurements (Supplementary Table 1). Thus, 10 synthetic heavy peptides, 5 in phosphorylated form and 5 in their matching unmodified peptide, were purchased in labeled with 13C/15 N on C-terminal lysine or arginine form from New England Peptide (Gardner, MA). Cysteines were modified by carbamidomethylation. Aliquots (30ul) of endogenous tryptic peptides were mixed with 30ul of synthetic peptides (at 24 nM concentration each). Details on the SRM method configuration are reported in Supplementary Table 2. We assessed the status of the following tau domains: AT8, AT100, 12E8, 77G7, and PHF-1 by quantifying the phosphorylation at the S202, T217, S262, S305 and S404 residues, respectively. To compensate for the changes of the abundance of the tau protein itself, the relative abundances of phosphopeptides were subtracted from the relative abundances of the corresponding non-modified peptides. Thus, the metric used in the downstream statistical analysis reflects the change in phosphorylation stoichiometry. Out of 982 older persons, 654 had available tau phosphopeptides’ data.

### Statistical analysis

We used Chi-square tests to examine the association between AWS and PWS watershed arteriolosclerosis, ANOVA to examine watershed arteriolosclerosis with age, and Wilcoxon rank sum tests to examine watershed arteriolosclerosis with β-amyloid burden and tau-tangle burden. Because the distribution of the measures of PHF-tau-tangle density was right skewed, we applied square root transformation and reduced skewness. Square root transformation was also applied to cortical β-amyloid load. Linear regression models adjusted for demographics and common age-related pathologies, including macroscopic and microscopic infarcts, atherosclerosis, CAA, Lewy bodies, and TDP-43, were used to examine associations between watershed arteriolosclerosis with cortical β-amyloid as the outcome and separately with tau tangles. We repeated the same linear regression models with regional β-amyloid and tau pathology burden as separate outcomes (mesial temporal and neocortical burden). Linear regression models adjusted for demographics, post-mortem interval (PMI), and other common age-related pathologies were used to examine associations of WMH with β-amyloid as the outcome, and separately with tau-tangle pathology. Finally, linear regression models adjusted for age-at-death, sex, education, and PMI were used to examine the association of anterior arteriolosclerosis and separately posterior watershed arteriosclerosis with change in tau phosphorylation stoichiometry. All analyses were conducted with SAS/STAT software version 9.4 (SAS Institute Inc, Cary, NC). Statistical significance was determined at nominal α level 0.05.

## Result

A total of 982 participants were included in the analyses. Mean age-at-death was 90 years with 69% being women. Characteristics of participants are presented in Table 1. Among those with dementia, 425 participants were diagnosed with Alzheimer’s dementia and 40 were diagnosed with vascular dementia. Among 982 participants, 22% older persons had diabetes, 16% were taking diabetes insulin medications, and 6% taking insulin injections. 70% of participants had history of hypertension and 79% were taking any anti-hypertensive medication. Smoking history was present in 33% of persons, with 31% being former smokers and 2% being current smokers. The mean systolic blood pressure across all visits was 132.7 (SD = 14.0) and the mean diastolic blood pressure across all visits was 72.5 (SD = 7.7). History of claudication was present in 31%, history of stroke in 20%, and history of myocardial infarction in 21%. Watershed arteriolosclerosis was common in the brain (Fig. 3), with moderate-to-severe arteriolosclerosis pathology being more frequent in the AWS than the PWS region (45% vs. 35% of older persons). Persons with higher severity of AWS arteriolosclerosis pathology were also likely to have higher severity of PWS arteriolosclerosis pathology (*X*^2^ = 251.97, degrees of freedom = 9, *p* < 0.001). Both AWS and PWS arteriolosclerosis pathologies were associated with age [(*F* (3, 978) = 8.28, *p* < 0.001 for AWS] and [*F* (3, 978) = 12.80, *p* < 0.001 for PWS].

**Table 1:** Characteristics of participants

	All (*n* = 982)
Demographics	
Age at death, mean y (SD)	90.3 (6.3)
Women, *n* (%)	683 (69.5%)
Education, mean (SD)	16.1 (3.5)
Clinical diagnosis	
No cognitive impairment	331 (33.7%)
Mild cognitive impairment	215 (21.9%)
Dementia	436 (44.4%)
Vascular risk factors, *N* (%)	
Diabetes	173 (22.5%)*
Hypertension	527 (68.6%)*
Neuropathologic, *N* (%)	
β-Amyloid burden, mean (SD)	1.6 (1.1)
Tau-tangle burden, mean (SD)	1.7 (1.5)
Moderate-to-severe AWS arteriolosclerosis	438 (44.6%)
Moderate-to-severe PWS arteriolosclerosis	341 (34.7%)
Moderate-to-severe CAA	354 (34.5%)*
Moderate-to-severe atherosclerosis	260 (26.5%)
Presence of microinfarcts	316 (32.2%)
Presence of macroscopic infarcts	351 (35.7%)
Moderate-to-severe TDP-43	329 (33.9%)*
Any Lewy bodies	258 (26.3%)*

**Data missing for *n* = 1 for diabetes; *n* = 1 for hypertension; *n* = 11 for CAA; *n* = 12 for TDP-43; *n* = 1 for Lewy body

### Watershed arteriolosclerosis and AD pathological changes

**Fig. 4:**
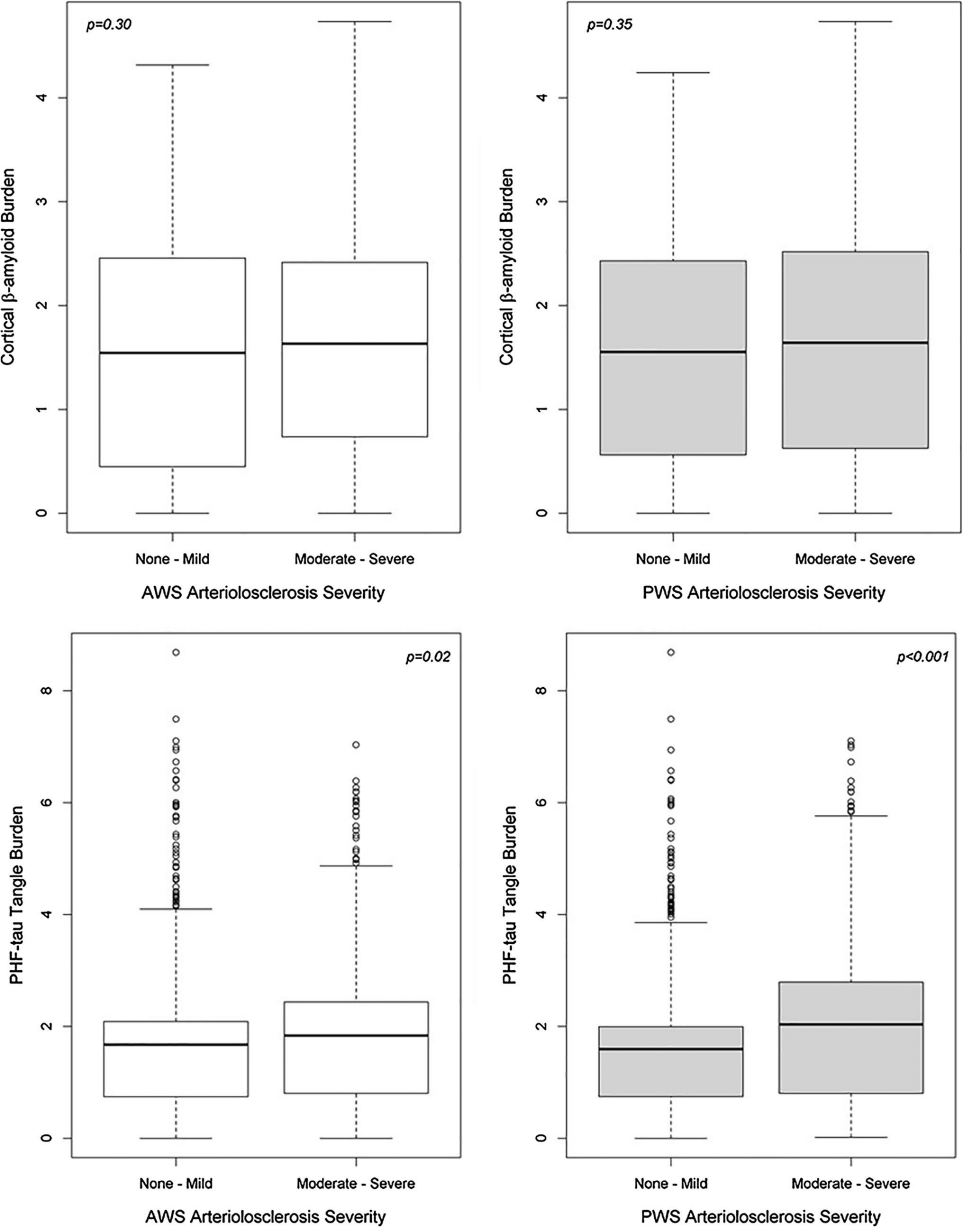
Cortical β-amyloid and tau-tangle burden across vessel severity. Boxplots of β-amyloid and tau-tangle burden across severity levels of AWS/PWS arteriolosclerosis pathology

**Fig. 5:**
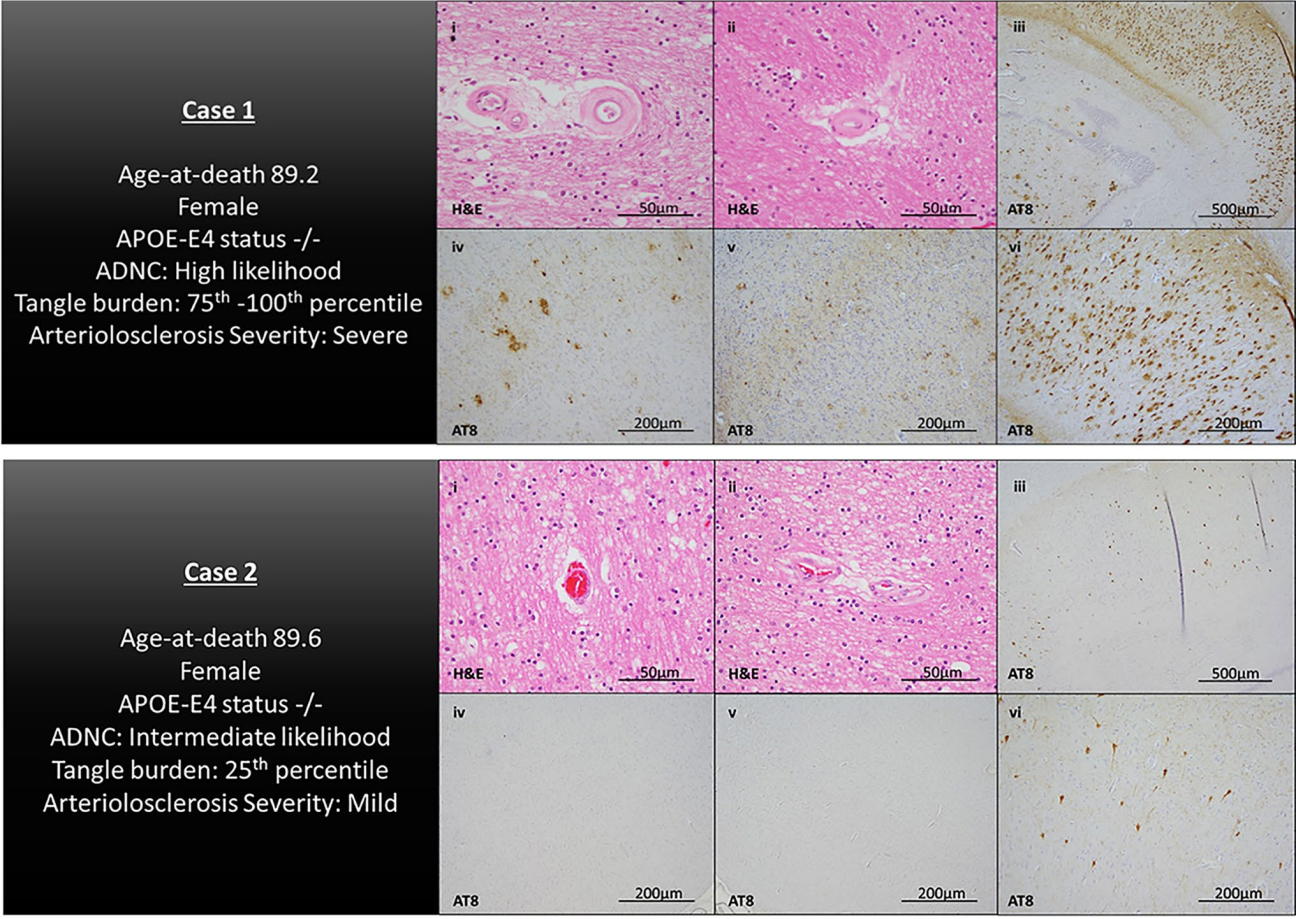
Illustrative cases. Histological sections from representative participants. Case 1 is a female participant, (age-at-death is 89.2 years), with a tau-tangle burden within the 75th–100th percentile and severe arteriolosclerosis pathology in watershed brain regions. Case 2 is a female participant (age-at-death is 89.6 years), with a tau-tangle burden within the 25th percentile and mild arteriosclerosis pathology in watershed brain regions. Images represent H&E-stained sections of the posterior watershed brain regions (i and ii) and AT8-stained sections for PHF-tau-tangle pathology in the CA1 subregion of the mid-hippocampus (iii and vi), midfrontal gyrus (iv), and inferior parietal cortex (v)

First, we examined the burden of β-amyloid and PHF-tau pathology across watershed vessel severity using Wilcoxon sum test. For persons with more severe arteriolosclerosis pathology in PWS region, PHF-tau-tangle burden was 40% higher than those with less severe vessel pathology (*p* < 0.001). For persons with more severe arteriolosclerosis pathology in AWS region, tau-tangle burden was 10% higher than those with less severe vessel pathology (*p* = 0.03) (Fig. 4). There was no significant difference between β-amyloid levels across AWS arteriolosclerosis severity (*p* = 0.30) or PWS arteriolosclerosis (*p* = 0.35). Histological sections showing arteriolosclerosis severity and tangle burden from age-matched participants are presented in Fig. 5.

Next, we examined the association of watershed arteriosclerosis in relation to AD pathology. In linear regression models adjusted for age, sex, education, and common age-related pathologies (CAA, atherosclerosis, macroscopic and microscopic infarcts, TDP-43, and Lewy body pathology), more severe PWS arteriolosclerosis pathology was associated with a higher burden of PHF-tau-tangle pathology. There was no significant association between PWS arteriolosclerosis and cortical β-amyloid or between AWS arteriolosclerosis and cortical β-amyloid/PHF-tau-tangle pathology (Table 2). Because both β-amyloid and tau-tangle pathology have stereotypical progression patterns in the brain, subsequent analyses examined whether associations were driven by regional burden; specifically, with mesial temporal and neocortical burden. Adjusted for demographics and common age-related pathologies, AWS arteriolosclerosis was not associated with neither mesial temporal nor neocortical β-amyloid/tau-tangle burden, while PWS arteriolosclerosis was associated with only neocortical tau-tangle burden (Table 2).

**Table 2:** Association watershed arteriolosclerosis with β-amyloid and tau-tangle pathology

	Overall amyloid	Mesial temporal β-amyloid	Neocortical β-amyloid	Overall tangle	Mesial temporal tau-tangle	Neocortical tau-tangle
AWS arteriolosclerosis	0.06 (0.04,0.13)	0.03 (0.03,0.32)	0.06 (0.04,0.12)	0.005 (0.04,0.91)	− 0.01 (0.06,0.87)	0.01 (0.05,0.67)
PWS arteriolosclerosis	− 0.04 (0.04, 0.29)	− 0.02 (0.03,0.59)	− 0.04 (0.04,0.28)	**0.10 (0.04, 0.01)**	0.09 (0.06,0.14)	**0.11 (0.04,0.008)**

Linear regression models with six separate outcomes, all adjusted for age-at-death, sex, education, CAA, atherosclerosis, macroscopic and microscopic infarcts, TDP-43, and Lewy body pathology. Values in cells are estimated coefficients (SE, *p* value)

*p*-values marked with bold indicate statistical significance

Prior studies have shown vascular risk factors to be associated with β-amyloid and tau burden. In sensitivity analyses, we further adjusted models for overall vascular risk burden (estimate = 0.11, SE = 0.04, *p* = 0.01), and in separate models, we adjusted for demographics and average systolic (estimate = 0.15, SE = 0.05, *p* = 0.001) and diastolic blood pressure (estimate = 0.15, SE = 0.05, *p* = 0.001). Notably, the association between PWS arteriolosclerosis and tangle burden in all sensitivity analyses remained unchanged. Using Spearman correlation, we further examined regional association between local arteriolosclerosis and local tau-tangle burden. We find that AWS arteriolosclerosis was not correlated with tangle burden in the midfrontal gyrus (rs = 0.05; *p* = 0.11), while PWS vessel disease was weakly correlated with cortical tau burden in both PWS region (rs = 0.10; *p* = 0.03) and angular gyrus (rs = 0.07; *p* = 0.02).

### Watershed arteriolosclerosis and tau protein

Because our primary findings revealed that watershed arteriolosclerosis pathology is associated with tau-tangle pathology, secondary analyses leveraged available tau proteomic data from the dorsolateral prefrontal cortex (*N* = 654) to examine the association between watershed arteriosclerosis and tau peptides. First, we examined Spearman correlations between each phosphorylated tau (ptau) epitope (S202, T217, S262, S305, S404) and tau-tangle pathology burden. The strongest correlations were the positive correlation between ptau S262 with tangle pathology (rs = 0.43; *p* < 0.001), as well as between ptau T217 and tangle pathology (rs = 0.35; *p* < 0.001). Next, in separate linear regression models adjusting for demographics and post-mortem interval (PMI), we examined whether AWS/PWS arteriolosclerosis was associated with each phosphorylated peptide. More severe PWS arteriolosclerosis pathology was associated with a higher abundance of all five phosphorylated tau peptides. Interestingly, we find AWS arteriolosclerosis pathology is associated with higher abundance of ptau S262 (Table 3).

**Table 3:** Association between watershed arteriolosclerosis and phosphorylated tau peptides

	S202	T217	S262	S305	S404
AWS arteriolosclerosis	0.01 (0.02, 0.71)	0.01 (0.03, 0.71)	**0.12 (0.06, 0.05)**	− 0.01 (0.05, 0.78)	− 0.01 (0.03, 0.78)
PWS arteriolosclerosis	**0.05 (0.02, 0.02)**	**0.07 (0.03, 0.03)**	**0.15 (0.06, 0.02)**	**0.13 (0.05, 0.006)**	**0.06 (0.03, 0.03)**

Linear regression models with change in tau phosphorylation stoichiometry as five separate outcomes, all adjusted for age-at-death, sex, education, and post-mortem interval. Values in cells are estimated coefficients (SE, *p* value).

*p*-values marked with bold indicate statistical significance

### Ex-vivo WMH and AD pathological changes

To translate our pathology findings to neuroimaging, we leveraged available whole brain measures of ex-vivo WMH data (*N* = 389). WMH lesions in aging have often been used as a surrogate marker for SVD in clinical studies and shown to be associated with arteriolosclerosis pathology in imaging-pathologic studies [2, 4]. Out of 389 participants, 19% had WMH burden = 1 (mild), 37% had WMH burden = 2 (moderate), and 44% had WMH burden = 3 (severe). In linear regression models adjusted for demographics, PMI, and common age-related pathologies, WMH burden was associated with greater tau-tangle pathology (estimate = 0.29, SE = 0.08, *p* = 0.001) but not with β-amyloid (estimate = 0.11, SE = 0.08, *p* = 0.19).

### Discussion

In the present study, we leveraged neuropathologic, neuroimaging, and proteomic data to examine the association between small vessel disease and AD pathological changes. Our main findings show that watershed arteriolosclerosis is associated with tau-tangle pathology and not β-amyloid. We extend these findings, indicating that watershed arteriosclerosis pathology is associated with proteomic tau phosphopeptides. Further, supporting these findings, we also show that whole brain measures of WMH burden, that are commonly used as a surrogate marker for clinical SVD, are associated with tau-tangle pathology. Together, these findings provide compelling evidence that SVD-related pathologic changes and tau are interconnected.

Watershed brain regions can provide a novel window into understanding the mechanisms associated with SVD. These brain regions have an idiosyncratic blood supply and can be considered as ‘weak points’ in the cerebral blood supply. We and others have shown watershed brain regions to be vulnerable to both hypoxic–ischemic-tissue injuries and arteriosclerotic vessel changes [1, 29]. Arteriolosclerosis is one of the most predominant SVD lesions co-existing with AD pathology in post-mortem brains [11]. Our current study extends these findings by showing that watershed arteriolosclerosis, specifically posterior watershed, is associated with greater tau-tangle pathology burden and higher abundance of tau phosphorylation epitopes. These findings are consistent with recent studies showing markers of SVD (both pathological and MRI) are associated with expression of tau pathological changes [31, 32, 34, 62]. To translate our findings to neuroimaging, we used WMH burden as a surrogate neuroimaging marker for SVD-related tissue injury. However, we recognize that while WMH are often presumed to be of vascular etiology, they are not specific to vascular disease, with their etiology most likely driven by a combination of both vascular and neurodegenerative factors. Notably, our findings between WMH and elevated tau burden corroborate other neuroimaging-pathological studies that report similar findings [24, 39]. Interestingly, though arteriosclerosis was more severe in the AWS compared to the PWS, it was largely the PWS arteriolosclerosis that we found an association with tau-tangle pathology, supporting the idea of brain vascular heterogeneity in the aging brain. The posterior watershed region presents as an interesting region. It is irrigated by terminal branches of all three anterior, middle, and posterior cerebral arteries, and overlaps specific brain regions (precuneus and posterior cingulate) susceptible to AD pathologic change [20, 21, 30, 49, 53]. We find an association between arteriosclerosis and neocortical tangle burden, suggesting that it is those persons with advanced tau pathologic changes that is driving the association, and suspect that this relationship is most prominent in symptomatic individuals. We did not find strong evidence of local SVD being associated with local tau pathology; however, further studies are needed to address whether watershed arteriolosclerosis is associated with tau burden among regionally or functionally connected brain regions. In contrast to other studies [19, 65, 66], we did not find an association of SVD (arteriolosclerosis or WMH) with β-amyloid burden. This may be due to study design as most of these studies have focused on neuroimaging markers of SVD, as well as PET markers for β-amyloid. While, PET ligands demonstrate excellent sensitivity and specificity to Aβ detection and have correlated well with the pathologic diagnosis of AD, these ligands are often used with a positive/negative threshold and cut-offs, which may not reflect diffuse Aβ positivity. Studies have shown that PET ligands have high specificity once Aβ adopts a beta-sheet fibrillar structure; therefore, a large proportion of PET amyloid is reflected by aggregated Aβ42, and therefore, PET binding to amyloid may be more prominent in neuritic plaques compared to diffuse plaques/vascular deposits [23].

Furthermore, various methodologies of quantifying amyloid (via PET/CSF/plasma) introduce differences across study design. For example, PET amyloid studies do not differentiate between parenchymal vs. vascular Abeta deposition, whereas our study included measures for meningeal and parenchymal CAA as confounding factors.

Typically, tau tangles accumulate in the entorhinal cortex and hippocampus followed by the neocortex. However, atypical AD pathologic subtypes have been described, where tau pathology spares the hippocampus [44]. Examining vascular changes in relation to neocortical tau in these atypical AD subtypes would be of interest. In addition, phosphorylated tau can also be detected in astrocyte glial cells and around the peri-vascular space, lesions often found in post-mortem brains with AD and other tauopathies [14, 33], raising the question of whether SVD differentially impacts tau pathognomonic lesions. Furthermore, supporting the hypothesis that SVD and tau are inter-related, neuropathologic studies have showed an association between SVD, white matter demyelination and Pick’s disease, and FTLD-tauopathy [62]. We and others have shown SVD pathology to be related to TDP-43 [1, 48], a proteinopathy frequently co-existing with AD pathology and associated with rapid cognitive decline and Alzheimer’s dementia in older persons [47]. While our data suggest that watershed arteriolosclerosis is associated with tau pathology independent of TDP-43, future work exploring key molecular vascular markers linked to TDP-43 encephalopathy, and more broadly to protein aggregation and proteotoxicity, will be important.

The exact mechanisms linking SVD and tau pathology are not well established. We conceptualize that SVD may be associated with tau phosphorylation through several downstream biological processes, including but not limited to neuroinflammation and oxidative stress associated with blood–brain barrier (BBB) dysfunction, which could impact key pathways that regulate intrinsic neuronal/cellular activity and function, thus leading to abnormal protein aggregation and neurodegeneration [52, 54]. Conversely, accumulation of dysfunctional tau protein may also lead to SVD-related lesions by directly impacting processes associated with vessel wall remodeling [42], as well as by altering white matter homeostasis [62]. Thus, resulting in a detrimental feedback loop mechanism between SVD and tau protein. One emerging topic in the field suggests that the glymphatic system represents a dynamic network to remove waste from the brain, including Aβ and tau [58, 69]. A myriad of biological processes including peri-vascular integrity, aquaporin-4 polarization, pericyte and endothelial cell organization, and inflammation within the vessel wall and within the peri-vascular space are important for facilitating glymphatic clearance. It is possible that morphological changes within the vessel due to the presence of arteriosclerosis can affect one/multiple biological processes necessary for conducting glymphatic clearance of Aβ and tau. In addition, studies have shown that neuroinflammation may lead to impaired glymphatic clearance [16]; therefore, it is possible that in the presence of heightened neuroinflammation-associated with AD pathology may impact the functioning of the glymphatic system and possibly exacerbate the frailty of this clearance system in the presence of severe vessel disease. Another intriguing topic is the tau propagation hypothesis which proposes a mechanism by which the pathological form of tau transfers between communicating neurons. The mechanisms associated with tau uptake in the extracellular space and subsequent intracellular uptake/aggregation are complex and many. It is possible that mechanisms of associated with SVD and tau propagation are linked. For example, in pathological conditions where morphological changes within the brain vasculature result in poor glymphatic clearance of tau from the extracellular space, it is likely that this would lead to intensifying tau propagation downstream. Furthermore, microglia may promote tau propagation via their ability to phagocytose and exocytose tau protein; thus, a heightened neuroinflammatory environment would also affect the vasculature and BBB function [14]. Altogether, the relationship between tau toxicity, SVD/vasculature, and neurodegeneration remains an interesting area and calls for the need of mechanistic vasculature studies.

It is well recognized that perfusion changes in persons with mid-cognitive impairment and Alzheimer’s dementia involve both temporal and parietal regions, with evidence to suggest that the earliest perfusion changes occur in the medial parietal cortex in persons with AD pathology [21, 43]. Interestingly, Neltner et al., showed that several medial temporal lobe regions can be vulnerable to arteriolosclerosis pathology, with the presence of arteriolosclerosis in temporal brain regions being greater in subjects with comorbid neurodegenerative pathologies [48]. These data directly align with our findings. We conceptualize that persons with higher levels of neurodegenerative proteinopathies (e.g., tau burden) may have more severe arteriosclerosis pathology in parietal and temporal regions, which may contribute to the hypoperfusion seen on neuroimaging studies in patients with Alzheimer’s dementia. However, further studies are needed to test whether SVD burden is associated with different aspects of neurodegeneration (e.g., neuronal death, inflammation, and brain atrophy).

Tau is a microtubule-associated protein, abundant in axons, and stabilizes microtubule bundles. For optimal function of tau, a normal level of phosphorylation is required. However, in pathological condition, an imbalance of phosphorylation events initiates abnormal metabolism and toxicity of tau. We find association of PWS arteriolosclerosis with dorsolateral prefrontal cortex expression of ptau Ser202, Thr217, Ser262, and Ser404, all of which are common tau epitopes phosphorylated in the AD brain [59]. Overall, phosphorylation of these tau epitopes changes the shape of tau molecule and modifies tau’s biological activity with microtubule bundles. Interestingly, arteriolosclerosis pathology in both AWS and PWS regions was associated with higher abundance of tau phosphorylation site Ser262, highlighting a more widespread role of arteriolosclerosis pathology with this specific tau epitope. Consistent with these findings, multiple animal studies also show an accumulation of Ser262 tau phosphopeptides after modeling ischemic stroke [7, 35, 36]. To enhance molecular understanding of AD pathophysiology, future studies examining AD-related proteomic changes in brain regions vulnerable to vascular changes, as well as angiogenesis/ischemic proteomic changes in regions vulnerable to early AD, are warranted.

Our findings raise the possibility that SVD plays a propagating role in tau pathologic change. It is well documented that β-amyloid drives tau accumulation leading to Alzheimer’s dementia [12, 64]; however, we speculate that tau represents a common pathway to dementia triggered by multiple factors, with one factor being SVD burden. Because mixed AD and cerebrovascular pathology account for over 80% of Alzheimer’s dementia diagnoses. Understanding these complex relationships between AD and vascular changes will be pivotal toward understanding AD pathophysiology and finding disease-modifying therapies.

There are multiple strengths to this study. By leveraging neuropathologic, ex-vivo neuroimaging, and proteomic, our study systematically identifies a relationship between SVD and tau pathologic change. We assessed pathology from watershed regions, providing important information on regional vascular vulnerability. However, we note several limitations. Although we examined two markers of SVD pathology (arteriolosclerosis and WMH), future work examining additional SVD markers (CAA, microbleeds, enlarged peri-vascular spaces) with AD and neurodegenerative proteinopathies in general is needed. There are no standard pathological consensus criteria assessing arteriolosclerosis pathology; future studies implementing a quantitative approach in evaluating brain arteriolosclerosis are needed. In addition, despite following standard protocols, we recognize that subtle differences with tissue sampling may occur which were not addressed in this study. Furthermore, we recognize that in the current study, tau phosphopeptides’ data were derived from the dorsolateral prefrontal cortex, a region involved later as per Braak staging, and we may be missing important molecular information if we were to examine proteomic changes in other AD vulnerable regions (e.g., hippocampus and precuneus). Finally, due to the cross-sectional nature of this study, we were unable to depict the underlying mechanisms of association of SVD and tau pathologic change.

## References

[1] Agrawal S, Yu L, Kapasi A, James BD, Arfanakis K, Barnes LL, Bennett DA, Nag S, Schneider JA (2021). Limbic-predominant age-related TDP-43 encephalopathy neuropathologic change and microvascular pathologies in community-dwelling older persons. Brain Pathol.

[2] Alosco ML, Sugarman MA, Besser LM, Tripodis Y, Martin B, Palmisano JN, Kowall NW, Au R, Mez J, DeCarli C, Stein TD, McKee AC, Killiany RJ, Stern RA (2018). A clinicopathological investigation of white matter hyperintensities and Alzheimer's disease neuropathology. J Alzheimers Dis.

[3] Andreev VP, Petyuk VA, Brewer HM, Karpievitch YV, Xie F, Clarke J, Camp D, Smith RD, Lieberman AP, Albin RL, Nawaz Z, El Hokayem J, Myers AJ (2012). Label-free quantitative LC-MS proteomics of Alzheimer's disease and normally aged human brains. J Proteome Res.

[4] Arfanakis K, Evia AM, Leurgans SE, Cardoso LFC, Kulkarni A, Alqam N, Lopes LF, Vieira D, Bennett DA, Schneider JA (2020). Neuropathologic correlates of white matter hyperintensities in a community-based cohort of older adults. J Alzheimers Dis.

[5] Arvanitakis Z, Capuano AW, Leurgans SE, Bennett DA, Schneider JA (2016). Relation of cerebral vessel disease to Alzheimer's disease dementia and cognitive function in elderly people: a cross-sectional study. Lancet Neurol.

[6] Arvanitakis Z, Capuano AW, Leurgans SE, Buchman AS, Bennett DA, Schneider JA (2017). The relationship of cerebral vessel pathology to brain microinfarcts. Brain Pathol.

[7] Basurto-Islas G, Gu JH, Tung YC, Liu F, Iqbal K (2018). Mechanism of tau hyperphosphorylation involving lysosomal enzyme asparagine endopeptidase in a mouse model of brain ischemia. J Alzheimers Dis.

[8] Bell RD, Zlokovic BV (2009). Neurovascular mechanisms and blood-brain barrier disorder in Alzheimer's disease. Acta Neuropathol.

[9] Bennett DA, Buchman AS, Boyle PA, Barnes LL, Wilson RS, Schneider JA (2018). Religious orders study and rush memory and aging project. J Alzheimers Dis.

[10] Bennett RE, Robbins AB, Hu M, Cao X, Betensky RA, Clark T, Das S, Hyman BT (2018). Tau induces blood vessel abnormalities and angiogenesis-related gene expression in P301L transgenic mice and human Alzheimer's disease. Proc Natl Acad Sci USA.

[11] Blevins BL, Vinters HV, Love S, Wilcock DM, Grinberg LT, Schneider JA, Kalaria RN, Katsumata Y, Gold BT, Wang DJJ, Ma SJ, Shade LMP, Fardo DW, Hartz AMS, Jicha GA, Nelson KB, Magaki SD, Schmitt FA, Teylan MA, Ighodaro ET, Phe P, Abner EL, Cykowski MD, Van Eldik LJ, Nelson PT (2021). Brain arteriolosclerosis. Acta Neuropathol.

[12] Bloom GS (2014). Amyloid-beta and tau: the trigger and bullet in Alzheimer disease pathogenesis. JAMA Neurol.

[13] Boyle PA, Yu L, Nag S, Leurgans S, Wilson RS, Bennett DA, Schneider JA (2015). Cerebral amyloid angiopathy and cognitive outcomes in community-based older persons. Neurology.

[14] Canepa E, Fossati S (2021). Impact of tau on neurovascular pathology in Alzheimer's disease. Front Neurol.

[15] Duncombe J, Kitamura A, Hase Y, Ihara M, Kalaria RN, Horsburgh K (2017). Chronic cerebral hypoperfusion: a key mechanism leading to vascular cognitive impairment and dementia. Closing the translational gap between rodent models and human vascular cognitive impairment and dementia. Clin Sci (Lond).

[16] Fukuda AM, Badaut J (2012). Aquaporin 4: a player in cerebral edema and neuroinflammation. J Neuroinflammation.

[17] Gouw AA, Seewann A, van der Flier WM, Barkhof F, Rozemuller AM, Scheltens P, Geurts JJ (2011). Heterogeneity of small vessel disease: a systematic review of MRI and histopathology correlations. J Neurol Neurosurg Psychiatry.

[18] Gurol ME, Biessels GJ, Polimeni JR (2020). Advanced neuroimaging to unravel mechanisms of cerebral small vessel diseases. Stroke.

[19] Hilal S, Akoudad S, van Duijn CM, Niessen WJ, Verbeek MM, Vanderstichele H, Stoops E, Ikram MA, Vernooij MW (2017). Plasma amyloid-beta levels, cerebral small vessel disease, and cognition: the Rotterdam study. J Alzheimers Dis.

[20] Hoenig MC, Bischof GN, Seemiller J, Hammes J, Kukolja J, Onur OA, Jessen F, Fliessbach K, Neumaier B, Fink GR, van Eimeren T, Drzezga A (2018). Networks of tau distribution in Alzheimer's disease. Brain.

[21] Huang CW, Hsu SW, Chang YT, Huang SH, Huang YC, Lee CC, Chang WN, Lui CC, Chen NC, Chang CC (2018). Cerebral perfusion insufficiency and relationships with cognitive deficits in alzheimer's disease: a multiparametric neuroimaging study. Sci Rep.

[22] Huang KL, Lin KJ, Ho MY, Chang YJ, Chang CH, Wey SP, Hsieh CJ, Yen TC, Hsiao IT, Lee TH (2012). Amyloid deposition after cerebral hypoperfusion: evidenced on [(18)F]AV-45 positron emission tomography. J Neurol Sci.

[23] Ikonomovic MD, Klunk WE, Abrahamson EE, Mathis CA, Price JC, Tsopelas ND, Lopresti BJ, Ziolko S, Bi W, Paljug WR, Debnath ML, Hope CE, Isanski BA, Hamilton RL, DeKosky ST (2008). Post-mortem correlates of in vivo PiB-PET amyloid imaging in a typical case of Alzheimer's disease. Brain.

[24] Jagust WJ, Zheng L, Harvey DJ, Mack WJ, Vinters HV, Weiner MW, Ellis WG, Zarow C, Mungas D, Reed BR, Kramer JH, Schuff N, DeCarli C, Chui HC (2008). Neuropathological basis of magnetic resonance images in aging and dementia. Ann Neurol.

[25] Jellinger KA, Attems J (2015). Challenges of multimorbidity of the aging brain: a critical update. J Neural Transm (Vienna).

[26] Kapasi A, Schneider JA (2016) Vascular contributions to cognitive impairment, clinical Alzheimer's disease, and dementia in older persons. Biochim Biophys Acta Doi:S0925-4439(15)00387-7 [pii]. 10.1016/j.bbadis.2015.12.023 PMC11062590 26769363

[27] Kapasi A, DeCarli C, Schneider JA (2017). Impact of multiple pathologies on the threshold for clinically overt dementia. Acta Neuropathol.

[28] Kapasi A, Leurgans SE, Arvanitakis Z, Barnes LL, Bennett DA, Schneider JA (2020) β-amyloid and tau tangle pathology modifies the association between small vessel disease and cortical microinfarcts. Stroke (**In Press**) 10.1161/STROKEAHA.120.031073 PMC7902459 33567873

[29] Kapasi A, Leurgans SE, James BD, Boyle PA, Arvanitakis Z, Nag S, Bennett DA, Buchman AS, Schneider JA (2018). Watershed microinfarct pathology and cognition in older persons. Neurobiol Aging.

[30] Karas G, Scheltens P, Rombouts S, van Schijndel R, Klein M, Jones B, van der Flier W, Vrenken H, Barkhof F (2007). Precuneus atrophy in early-onset Alzheimer's disease: a morphometric structural MRI study. Neuroradiology.

[31] Kester MI, Goos JD, Teunissen CE, Benedictus MR, Bouwman FH, Wattjes MP, Barkhof F, Scheltens P, van der Flier WM (2014). Associations between cerebral small-vessel disease and Alzheimer disease pathology as measured by cerebrospinal fluid biomarkers. JAMA Neurol.

[32] Kim HJ, Park S, Cho H, Jang YK, San Lee J, Jang H, Kim Y, Kim KW, Ryu YH, Choi JY, Moon SH, Weiner MW, Jagust WJ, Rabinovici GD, DeCarli C, Lyoo CH, Na DL, Seo SW (2018). Assessment of extent and role of tau in subcortical vascular cognitive impairment using 18F-AV1451 positron emission tomography imaging. JAMA Neurol.

[33] Kovacs GG (2020). Astroglia and tau: new perspectives. Front Aging Neurosci.

[34] Laing KK, Simoes S, Baena-Caldas GP, Lao PJ, Kothiya M, Igwe KC, Chesebro AG, Houck AL, Pedraza L, Hernandez AI, Li J, Zimmerman ME, Luchsinger JA, Barone FC, Moreno H, Brickman AM, Initiative ADN (2020). Cerebrovascular disease promotes tau pathology in Alzheimer's disease. Brain Commun.

[35] Mailliot C, Podevin-Dimster V, Rosenthal RE, Sergeant N, Delacourte A, Fiskum G, Buee L (2000). Rapid tau protein dephosphorylation and differential rephosphorylation during cardiac arrest-induced cerebral ischemia and reperfusion. J Cereb Blood Flow Metab.

[36] Majd S, Power JHT, Koblar SA, Grantham HJM (2016). The impact of tau hyperphosphorylation at Ser(262) on memory and learning after global brain ischaemia in a rat model of reversible cardiac arrest. IBRO Rep.

[37] Mangla R, Kolar B, Almast J, Ekholm SE (2011). Border zone infarcts: pathophysiologic and imaging characteristics. Radiographics.

[38] Marchant NL, Reed BR, DeCarli CS, Madison CM, Weiner MW, Chui HC, Jagust WJ (2012). Cerebrovascular disease, beta-amyloid, and cognition in aging. Neurobiol Aging.

[39] McAleese KE, Walker L, Graham S, Moya ELJ, Johnson M, Erskine D, Colloby SJ, Dey M, Martin-Ruiz C, Taylor JP, Thomas AJ, McKeith IG, De Carli C, Attems J (2017). Parietal white matter lesions in Alzheimer's disease are associated with cortical neurodegenerative pathology, but not with small vessel disease. Acta Neuropathol.

[40] McDade E, Kim A, James J, Sheu LK, Kuan DC, Minhas D, Gianaros PJ, Ikonomovic S, Lopez O, Snitz B, Price J, Becker J, Mathis C, Klunk W (2014). Cerebral perfusion alterations and cerebral amyloid in autosomal dominant Alzheimer disease. Neurology.

[41] McKeith IG, Galasko D, Kosaka K, Perry EK, Dickson DW, Hansen LA, Salmon DP, Lowe J, Mirra SS, Byrne EJ, Lennox G, Quinn NP, Edwardson JA, Ince PG, Bergeron C, Burns A, Miller BL, Lovestone S, Collerton D, Jansen EN, Ballard C, de Vos RA, Wilcock GK, Jellinger KA, Perry RH (1996). Consensus guidelines for the clinical and pathologic diagnosis of dementia with Lewy bodies (DLB): report of the consortium on DLB international workshop. Neurology.

[42] Merlini M, Wanner D, Nitsch RM (2016). Tau pathology-dependent remodelling of cerebral arteries precedes Alzheimer's disease-related microvascular cerebral amyloid angiopathy. Acta Neuropathol.

[43] Miners JS, Palmer JC, Love S (2015). Pathophysiology of hypoperfusion of the precuneus in early Alzheimer's disease. Brain Pathol.

[44] Murray ME, Cannon A, Graff-Radford NR, Liesinger AM, Rutherford NJ, Ross OA, Duara R, Carrasquillo MM, Rademakers R, Dickson DW (2014). Differential clinicopathologic and genetic features of late-onset amnestic dementias. Acta Neuropathol.

[45] Nag S, Yu L, Boyle PA, Leurgans SE, Bennett DA, Schneider JA (2018). TDP-43 pathology in anterior temporal pole cortex in aging and Alzheimer's disease. Acta Neuropathol Commun.

[46] Nelson AR, Sweeney MD, Sagare AP, Zlokovic BV (2015) Neurovascular dysfunction and neurodegeneration in dementia and Alzheimer's disease. Biochim Biophys Acta Doi:S0925-4439(15)00370-1 10.1016/j.bbadis.2015.12.016 PMC4821735 26705676

[47] Nelson PT, Dickson DW, Trojanowski JQ, Jack CR, Boyle PA, Arfanakis K, Rademakers R, Alafuzoff I, Attems J, Brayne C, Coyle-Gilchrist ITS, Chui HC, Fardo DW, Flanagan ME, Halliday G, Hokkanen SRK, Hunter S, Jicha GA, Katsumata Y, Kawas CH, Keene CD, Kovacs GG, Kukull WA, Levey AI, Makkinejad N, Montine TJ, Murayama S, Murray ME, Nag S, Rissman RA, Seeley WW, Sperling RA, White Iii CL, Yu L, Schneider JA (2019). Limbic-predominant age-related TDP-43 encephalopathy (LATE): consensus working group report. Brain.

[48] Neltner JH, Abner EL, Baker S, Schmitt FA, Kryscio RJ, Jicha GA, Smith CD, Hammack E, Kukull WA, Brenowitz WD, Van Eldik LJ, Nelson PT (2014). Arteriolosclerosis that affects multiple brain regions is linked to hippocampal sclerosis of ageing. Brain.

[49] Palmqvist S, Scholl M, Strandberg O, Mattsson N, Stomrud E, Zetterberg H, Blennow K, Landau S, Jagust W, Hansson O (2017). Earliest accumulation of beta-amyloid occurs within the default-mode network and concurrently affects brain connectivity. Nat Commun.

[50] Petyuk VA, Qian WJ, Smith RD, Smith DJ (2010). Mapping protein abundance patterns in the brain using voxelation combined with liquid chromatography and mass spectrometry. Methods.

[51] Rabin JS, Yang HS, Schultz AP, Hanseeuw BJ, Hedden T, Viswanathan A, Gatchel JR, Marshall GA, Kilpatrick E, Klein H, Rao V, Buckley RF, Yau WW, Kirn DR, Rentz DM, Johnson KA, Sperling RA, Chhatwal JP (2018). Vascular risk and beta-amyloid are synergistically associated with cortical tau. Ann Neurol.

[52] Raz L, Bhaskar K, Weaver J, Marini S, Zhang Q, Thompson JF, Espinoza C, Iqbal S, Maphis NM, Weston L, Sillerud LO, Caprihan A, Pesko JC, Erhardt EB, Rosenberg GA (2019). Hypoxia promotes tau hyperphosphorylation with associated neuropathology in vascular dysfunction. Neurobiol Dis.

[53] Scheff SW, Price DA, Ansari MA, Roberts KN, Schmitt FA, Ikonomovic MD, Mufson EJ (2015). Synaptic change in the posterior cingulate gyrus in the progression of Alzheimer's disease. J Alzheimers Dis.

[54] Scheffer S, Hermkens DMA, van der Weerd L, de Vries HE, Daemen MJAP (2021). Vascular hypothesis of Alzheimer disease: topical review of mouse models. Arterioscler Thromb Vasc Biol.

[55] Schneider JA, Boyle PA, Arvanitakis Z, Bienias JL, Bennett DA (2007). Subcortical infarcts, Alzheimer's disease pathology, and memory function in older persons. Ann Neurol.

[56] Schneider JA, Arvanitakis Z, Yu L, Boyle PA, Leurgans SE, Bennett DA (2012). Cognitive impairment, decline and fluctuations in older community-dwelling subjects with Lewy bodies. Brain.

[57] Schneider JA, Wilson RS, Cochran EJ, Bienias JL, Arnold SE, Evans DA, Bennett DA (2003). Relation of cerebral infarctions to dementia and cognitive function in older persons. Neurology.

[58] Silva I, Silva J, Ferreira R, Trigo D (2021). Glymphatic system, AQP4, and their implications in Alzheimer's disease. Neurol Res Pract.

[59] Simic G, Babic Leko M, Wray S, Harrington C, Delalle I, Jovanov-Milosevic N, Bazadona D, Buee L, de Silva R, Di Giovanni G, Wischik C, Hof PR (2016). Tau protein hyperphosphorylation and aggregation in Alzheimer's disease and other tauopathies, and possible neuroprotective strategies. Biomolecules.

[60] Strozyk D, Dickson DW, Lipton RB, Katz M, Derby CA, Lee S, Wang C, Verghese J (2010). Contribution of vascular pathology to the clinical expression of dementia. Neurobiol Aging.

[61] Suter OC, Sunthorn T, Kraftsik R, Straubel J, Darekar P, Khalili K, Miklossy J (2002). Cerebral hypoperfusion generates cortical watershed microinfarcts in Alzheimer disease. Stroke.

[62] Thal DR, von Arnim CA, Griffin WS, Mrak RE, Walker L, Attems J, Arzberger T (2015). Frontotemporal lobar degeneration FTLD-tau: preclinical lesions, vascular, and Alzheimer-related co-pathologies. J Neural Transm (Vienna).

[63] Utter S, Tamboli IY, Walter J, Upadhaya AR, Birkenmeier G, Pietrzik CU, Ghebremedhin E, Thal DR (2008). Cerebral small vessel disease-induced apolipoprotein E leakage is associated with Alzheimer disease and the accumulation of amyloid beta-protein in perivascular astrocytes. J Neuropathol Exp Neurol.

[64] van der Kant R, Goldstein LSB, Ossenkoppele R (2020). Amyloid-beta-independent regulators of tau pathology in Alzheimer disease. Nat Rev Neurosci.

[65] van Leijsen EMC, Kuiperij HB, Kersten I, Bergkamp MI, van Uden IWM, Vanderstichele H, Stoops E, Claassen JAHR, van Dijk EJ, de Leeuw FE, Verbeek MM (2018). Plasma abeta (Amyloid-beta) levels and severity and progression of small vessel disease. Stroke.

[66] van Westen D, Lindqvist D, Blennow K, Minthon L, Nagga K, Stomrud E, Zetterberg H, Hansson O (2016). Cerebral white matter lesions—associations with Abeta isoforms and amyloid PET. Sci Rep.

[67] Vemuri P, Lesnick TG, Przybelski SA, Knopman DS, Preboske GM, Kantarci K, Raman MR, Machulda MM, Mielke MM, Lowe VJ, Senjem ML, Gunter JL, Rocca WA, Roberts RO, Petersen RC, Jack CR (2015). Vascular and amyloid pathologies are independent predictors of cognitive decline in normal elderly. Brain.

[68] Wilcock DM, Schmitt FA, Head E (2015) Cerebrovascular contributions to aging and Alzheimer's disease in Down syndrome. Biochim Biophys Acta Doi:S0925-4439(15)00337-3 [pii]. 10.1016/j.bbadis.2015.11.007 PMC4821721 26593849

[69] Wu J, Carlock C, Shim J, Moreno-Gonzalez I, Glass W, Ross A, Barichello T, Quevedo J, Lou Y (2021). Requirement of brain interleukin33 for aquaporin4 expression in astrocytes and glymphatic drainage of abnormal tau. Mol Psychiatry.

[70] You Y, Perkins A, Cisternas P, Munoz B, Taylor X, You Y, Garringer HJ, Oblak AL, Atwood BK, Vidal R, Lasagna-Reeves CA (2019). Tau as a mediator of neurotoxicity associated to cerebral amyloid angiopathy. Acta Neuropathol Commun.

[71] Yu L, Petyuk VA, Gaiteri C, Mostafavi S, Young-Pearse T, Shah RC, Buchman AS, Schneider JA, Piehowski PD, Sontag RL, Fillmore TL, Shi T, Smith RD, De Jager PL, Bennett DA (2018). Targeted brain proteomics uncover multiple pathways to Alzheimer's dementia. Ann Neurol.

